# Medical Intelligence

**Published:** 1857-03

**Authors:** 


### MEDICAL INTELLIGENCE.

*Rush Medical College. Public Annual Commencement of Session for* 1856–7.

The public exercises attendant on the close of the regular annual College term, took place in the Hall of the College on the 18th of February, and were attended by a large and attentive audience. The exercises commenced at 2½ o'clock P.M., and were opened with prayer by the Rev. Dr. Barker. Several of the candidates for graduation were then required to read their theses, after which the Degree of Doctor of Medicine was cônferred by Prof. D. Brainard, President of the College, on forty-one members of the class, who had previously passed a rigid examination and been recommended to the Board of Trustees. The Honorary Degree of M.D. was also conferred on Dr. William Long, of New Maysville, Indiana; and on Dr. H. Noble, of McLean County, Illinois. An *ad eundem* Degree was, at the same time, conferred on Dr. J. W. York, of Shelby Co. Illinois. The Valedictory Address was then delivered by Prof. Brainard, in his usual impressive and happy manner. His theme was the 'Ethics and Etiquette of the Profession,'' than which no one more appropriate could have been chosen. His mode of presenting it was such as to impress important practical rules and precepts strongly upon the minds of his hearers. After the address and benediction, the further proceedings are described as follows in the *Daily Democratic Press*, published the following morning, viz:—

'After the delivery of the Valedictory, the Professors, Trustees, Students, and guests were invited to the entertainment provided by the Faculty in the Museum Hall, where a substantial repast was in waiting for them. That over, the graduates called the meeting to order, when the following toasts were offered and responded to:—

"1. The Chair of Surgery—Its eloquence our delight; its scientific truths our inheritance; its investigations an invaluable addition to the science of surgery. May its memory never fade."

'Dr. Brainard, Professor of Surgery, responded in an able speech.

"2. The Chair of Physiology, and its present worthy occupant, Prof. W. B. Herrick."

'Dr. Herrick was called for, but he was not in the hall.

"3. The Chair of Chemistry—Professor Blaney, the Liebig of America."

'Responded to by Professor Blaney in his usual happy manner.

"4. The Chair of Obstetrics and its worthy occupant—When he retires, may his well-known piety and virtue be as great a blessing to the world as his attainments have been a boon to science."

'Prof. J. Evans responded in a touching speech, announcing it as his intention to retire from the professorship as soon as his colleagues appointed a successor.

"5. Prof. N. S. Davis, the Working Man."

'Responded to by Dr. Davis in an excellent speech, indicating industry and labor as the physician's only ladder to honorable eminence.

"6. Prof. H. A. Johnson—a brother, who recognizes the same Alma Mater—may he live to reflect credit to it and us by his eminent scientific attainments."

'Dr. Johnson, Professor of Materia Medica, responded in a neat and appropriate speech.

"7. Prof. J. W. Freer—the bone and sinew of the Faculty."

'Dr. Freer, Professor of Anatomy, amid bursts of laughter, made a humorous reply.

"8. Dr. Hollister, our able Demonstrator of Anatomy—he has made the dead to rise."

'Dr. Hollister responded.

'Professor Brainard then gave the following:—

"The Graduating Class. May their future career be honorable as their past has been successful.

'Dr. Wardner being called out responded.

"The Under-Graduates—the Embryo-Graduates of the next year."

'Responded to by Mr. Swafford.

'A Graduate gave, "Rush Medical College—may it always stand."

'Prof. Evans offered, "The Press"—which was responded to by J. F. Ballantyne, of the *Democratic Press*.

'One of the graduates sung "Napoleon's Grave," in an able manner, after which the meeting dispersed.

'Rush Medical College, through the zeal and industry of its Faculty, is an institution of which Chicago may well feel proud. Though comparatively young, it has attained a position in the science of medicine second to few Universities in the United States. Its professors show by their laudable zeal and energy that they are determined to render it an ornament to the profession, as well as an instrument for advancing scientific knowledge in the West and North-West.'

Without boasting, we may claim that few medical classes in this or any other country have enjoyed better advantages for attaining knowledge, than the one in attendance on Rush Medical College during the past session. For, besides the ordinary College course, in which the *material* for dissection and the means of illustration in the several departments were ample, the class had easy access to two Hospitals well filled with patients. In one, regular clinics were given on *four* mornings of each week, and in the other, *six* mornings; thereby enabling the class taking the Hospital tickets to be so divided as to allow the most direct and free examination of patients. So complete were these clinical arrangements that scarcely a physical sign could be named, arising from acute and chronic diseases affecting the viscera of the chest, which was not carefully examined and listened to personally, during some part of the term, by the 72 members of the class, who attended the medical wards of the Mercy Hospital; while the opportunities for studying both operative surgery and surgical diseases, such as opthalmia, syphilis, ulcers, &c., under Prof. Brainard in the Marine, and Prof. Freer in the Surgical Wards of the Mercy Hospitals, were as ample as could be desired. The following is a list of the regular graduates, with the titles to their Inaugural Theses, viz:—

LIST OF GRADUATES.

'James Franklin Cravens, Chicago, "Mechanism of Labor."

'James Francis Marrh, Chicago—"Treatment of Simple Fractures by Starch Bandages."

'David Hewitt Spickler, State Line, Pa.—"Bronchitis."

'Charles Hammel, Ottawa, Ill.—"Scarlatina."

'John Jasper Lake, Mount Pleasant, De Witt Co.—"Typhoid Fever."

'Benjamin Wilson, Chambersburg, Ill.—"Scarlatina."

'Joseph Thomas Miller, Urbana, Ill.—"Combustion."

'M. H. Bounel, Yountsville, Ind.—"Dysentery."

'Edward A. Wilcox, Lacon, Ill.—"Cause of Intermittents."

'A. M. D. Hughes, Payson, Ill.—"Blood-Letting."

'J. B. Paul, Havana, Ill.—"Intestinal Diseases—Causes and Treatment."

'E. F. Hubbard, Wauconda, Ill.—"Aliments as Therapeutical Agents."

'Samuel Higinbotham, Plymouth, Ill.—"The Reparative Process."

'P. J. Wardner, Chicago, Ill.—"Deposits of Earthy Phosphates in Urine."

'Edwin Powell, Chicago, Ill.—"Pneumonia."

'Lafayette H. Smith, Chicago, Ill.—"Syphilis."

'A. W. Adair, Coatsville, Ind.—"Veratrum Viride."

'T. A. Graham, Clermont, Ind.—"Acute Rheumatism."

'T. D. Fisher, Leroy, Ill.—"Water a Therapeutic Agent."

'A. L. Kimber, Prairie City, Ill.—"Pathology of Dropsy."

'D. C. Bennett, Janesville, Wis.—"Inflammation."

'John C. Lowrie, Chicago, Ill.—'Gonorrhœa."

'Geo. W. Wilkinson, Kickapoo, Ill.—"Etiology of Milk Sickness."

'J. P. Terrell, Huntingdon, Ind.—"Medical Properties of Iodine of Potassa."

'Stephen L. Urmston, Iroquois, Ill.—"Selection of Remedies."

'Thos. B. Dever, Lacon, Ill.—"Erysipelas."

'N. O. Pearson, Palestine, Ill.—"Duties of a Surgeon before Operating."

'L. H. Dunn, Tiskilwa, Ill.—"Venesection."

'W. M. Hall, Walker's Grove, Ill.—"Intermittent Fever."

'Josiah L. Philips, Farmington, Me.—"Suppuration."

'J. Sumner Bowen, Roanoke, Ind.—"Digestion."

'Wm. F. Vermilion, Martinsville, Ill.—"Dysentery."

'Thos. J. Shreves, Avon, Ill.—"Typhoid Fever."

'Benj. Woodward, Sharon, Ill.—"The Physician—his Qualifications and Mission."

'John H. Tyler, DeWitt Co., Ill.—"Diagnosis."

'James McCleery, Monroe, Jasper Co., Iowa.—"Duties and Qualifications of the Physician."

'Francis W. White, Chicago, Ill.—"Pathology of Rheumatic Fever."

'Benj. F. White, Two Rivers, Wis.—"Preliminary Qualifications Necessary for the Study of Medicine."

'Charles Hill, Lebanon, Ill.—"Hygienic Agents."

'Ethan McAferty, Lexington, Ill.—"Erysipelas."

'Lafayette Gray, Ipava, Ill.—"Generation, Conception, &c."'

